# Telecom Light-Emitting Diodes Based on Nanoconfined Self-Assembled Silicon-Based Color Centers

**DOI:** 10.1021/acsphotonics.4c01662

**Published:** 2025-05-07

**Authors:** Andreas Salomon, Johannes Aberl, Enrique Prado Navarrete, Merve Karaman, Oliver E. Lang, Daniel Primetzhofer, Peter Deák, Ádám Gali, Thomas Fromherz, Moritz Brehm

**Affiliations:** a Institute of Semiconductor and Solid State Physics, Johannes Kepler University, Altenberger Straße 69, Linz 4040, Austria; b Department of Physics and Astronomy, Uppsala University, Box 516, Uppsala 75120, Sweden; c HUN-REN Wigner Research Centre for Physics, P.O. Box 49, Budapest H-1525, Hungary; d Beijing Computational Science Research Center Beijing 100193, China; e Department of Atomic Physics, Institute of Physics, Budapest University of Technology and Economics, Műegyetem rakpart 3., Budapest H-1111, Hungary; f MTA-WFK Lendület “Momentum” Semiconductor Nanostructures Research Group, P.O. Box 49, Budapest H-1525, Hungary

**Keywords:** silicon, epitaxy, self-assembly, electroluminescence, point-defects

## Abstract

Silicon color centers (SiCCs) have recently emerged as potential building blocks for light emitters in Si photonics, quantum emitters with spin storage capabilities, and Si-based quantum repeaters. We have recently developed a noninvasive method to engineer carbon-related SiCCs confined to ultrathin nanolayers within a pristine crystalline environment, which is of utmost importance for the photostability of SiCCs. Here, we demonstrate embedding these C-doping-based SiCCs into the only 9 nm wide intrinsic region of a p-i-n diode using the epitaxial self-assembly of color centers. We report electrically pumped light emission with an exponential increase in the intensity as a function of the driving current until saturation. We associate this property with the shift of quasi-Fermi-level position upon electrical driving, which simultaneously improves the spectral homogeneity of the engineered SiCCs. Despite the low employed growth temperatures, our study demonstrates the electrical control and driving of near-infrared emitters in high-quality silicon diodes, an essential milestone for advancing classical and quantum optoelectronics.

## Graphical abstract

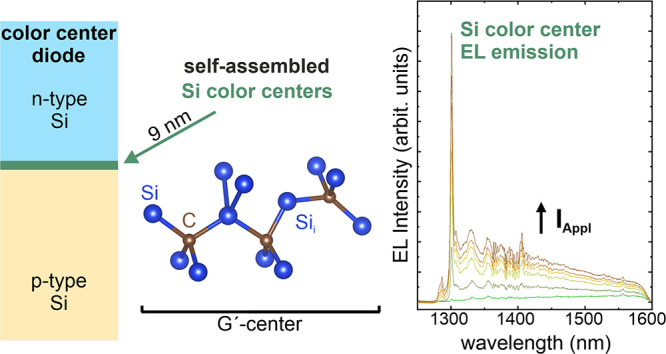

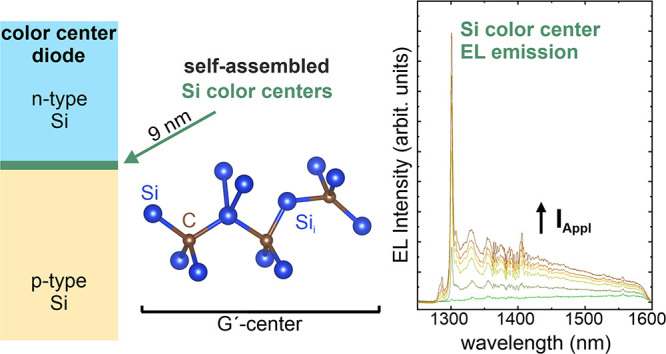

## Introduction

Silicon color centers (SiCCs) have been studied since the 70s as detrimental side effects caused by radiation damage in the fabrication of microelectronic devices.^1^ However, recently, they experienced a renaissance due to their peculiar light emission properties. On the one hand, SiCCs such as the so-called G-center, a carbon-based point defect composed of two substitutional carbon atoms and one interstitial Si atom,^2,3^ have been shown to provide optical gain^3^ for potential lasing applications in the telecom spectral range.^3,4^ In that respect, efficient Si-based light emission for optical interconnects still remains a missing piece and represents the “holy grail” of silicon photonics.^5–9^ Thereby, in contrast to III–V lasers on Si,^7–9^ the light emission of Si color centers is restricted to cryogenic temperatures only. However, optical interface applications between cryogenic electronics^10^ and quantum electronics can be envisioned. On the other hand, several types of isolated SiCCs, including single G-centers, W-centers, or T-centers have recently exhibited promising quantum properties such as high-purity single photon emission and spin manipulation capabilities.^11–15^ In particular, the G-center could serve as a potential quantum memory that can be optically read out at telecom wavelength^2,16,17^ that can particularly benefit from the CC’s compatibility with Si and SOI integration technology. However, for both, SiCC-based cryo-lasers and quantum-emitters, embedding them into a p-(i)-n diode is of key importance, which has been demonstrated previously.^18–25^ Useful integrated lasers must be driven electrically,^7–9^ and diodes operated in reverse bias conditions are used to clear the charge environment and limit blinking and spectral diffusion of quantum emitters.^26^

Conventionally, however, CC creation is enabled by single- or multistep ion implantation. For example, for the fabrication of G-centers on Si or SOI, implantation energies for C ions >10 keV are used,^11–13^ which, on the one hand, provide the targeted stopping range for color center emission but, on the other hand, lead to a Gaussian distribution profile for SiCC creation that spreads throughout more than hundred nm (see Figure 1(a)).^27,28^ This spread leads to intrinsic constraints in photonic device designs, i.e., photonic mode – emitter coupling and variations among device ensembles. Implantation at lower energies could, in principle, limit the emitter spread but leads to the creation of emitters close to the surface. Surfaces and interfaces are always troublesome regarding their interaction with the (quantum)-emitters.^29^ Compared to SiCCs that are excited optically, the emitter spread is typically even worse for electrically pumped devices since the implantation occurs through thick top-contact layers,^19–21,27^ which calls for higher implantation energies and thus leads to an even larger emitter spread see Figure 1(a). Note that the emitter spread worsens further if channeling effects are present during the ion implantation. A method that reduces this effect was demonstrated recently using lateral diodes.^18^ However, lateral diodes that underwent ion implantation for CC-formation are still subjected to the same vertical emitter distribution problem as optically pumped samples.

**Figure 1:**
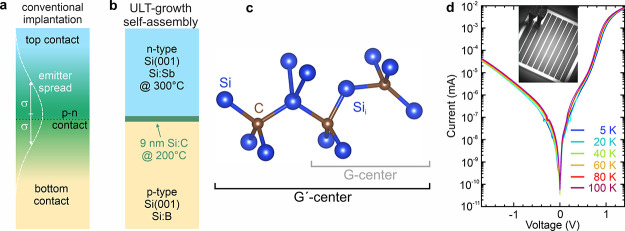
Growth scheme and diode characteristics. (a) Typical fabrication scheme of group-IV-based color centers embedded in vertical p-n diodes. The implantation through the top contact leads to a wide emitter spread after implantation. (b) Si color center light emitting diode fabricated entirely without ion implantation. The SiCCs result from self-assembly during the growth of C-doped Si at ultralow growth temperatures of 200 °C. The n-type top contact was grown at a low enough growth temperature to avoid the dissociation of the SiCCs in the underlying thin nanolayer. (c) Atomistic model of the G’-center, consisting of a G-center and an adjacent substitutional C atom. (d) Temperature-dependent I–V characteristics of the fabricated device, indicating diode behavior. The inset shows a photograph of the device under test.

Here, we demonstrate that carbon-based SiCC light-emitting diodes (LEDs) can be fabricated without ion implantation by solely using epitaxy for the formation of the p-n diode as well as the CCs. Importantly, the CCs can be formed deterministically in nanometer-thin layers of C-doped Si that can be grown exactly in the ultrathin intrinsic region of the p-i-n structure of the diode.

We emphasize that the top contact formation after the CC emitter creation asks for cautiousness regarding the employed growth conditions since SiCCs are fragile with respect to thermal annealing, i.e., G-centers, and W-centers annihilate already at annealing temperatures ranging from 200 to 300 °C.^1^ Ultralow growth temperatures (T_S_ ≤ 300 °C) and excellent growth pressures are needed to (i) enable the *in situ* formation of SiCCs with excellent optical properties, (ii) avoid SiCC dissociation during top contact growth, maintaining a high crystalline quality, and (iii) enable an optimal device functionality with an emission that is not inferior as compared to optically pumped samples. We show that despite the low required growth temperatures, epitaxial growth of diodes is feasible, and we demonstrate pronounced CC emission at low current densities <1 A/cm^2^ from a SiCC layer as thin as 9 nm located at the p–n junction of the diode. Thus, we present a proof-of-concept fabrication method demonstrating that electrically driven SiCCs can be deterministically confined down to layer thicknesses less than 10 nm, opening up the path for potential applications as light sources in Si-based photonics and scalable integrated quantum technology. Furthermore, we show that the present electric field can lead to an improved spectral homogeneity of the engineered SiCCs.

## Materials and Methods

### Color Center Self-Assembly

The samples for electroluminescence (EL) investigations were grown on Czochralski, p-type, B-doped 4-in. Si(001) substrates (0.001–0.005 Ωcm) using solid source molecular beam epitaxy in a Riber SIVA-45 chamber. The doped substrate provides the p-region of the p-i-n diode. The base pressure in the chamber was 5 × 10^–11^ mbar, and the maximum pressure during the growth was 3 × 10^–10^ mbar. The growth protocol can be found in Figure S1 of the Supporting Information. The samples for photoluminescence (PL) characterization were grown on FZ Si(001) wafers with resistivities >5000 Ωcm. All substrates were cleaned using conventional Si cleaning, see ref.^30^ After loading the samples into the MBE chamber, they were initially degassed in an ultrahigh vacuum at 700 °C for 15 min. Hereafter, the samples were kept at 450 °C for 30 min. Initially, a p-type, B-doped Si buffer layer with a doping concentration of 5 × 10^18^ cm^–3^ and a thickness of 50 nm was grown at T_S_ = 500 °C. After that, T_S_ was ramped down to 200 °C, the growth temperature of the C-doped Si layer. At a C-doping concentration of 3.8 × 10^19^ cm^–3^, a 9 nm thick Si:C layer was deposited. Thereafter, T_S_ was ramped to 300 °C, at which the n-type top contact of the diode was grown. It consisted of a 50 nm thick Si:Sb layer with a doping concentration of 5 × 10^18^ cm^–3^, followed by a 150 nm thick Si:Sb layer with a doping concentration of 2·10^19^ cm^–3^, leading to symmetric doping concentrations around the intrinsic Si:C layer hosting the CCs. As a reference, an all Si diode was grown, for which the Si:C layer was replaced by a 9 nm thick intrinsic Si layer, deposited at T_S_ = 310 °C. For the complementary PL investigations of the C-doping concentration dependence on the luminescence properties of the SiCCs, several additional reference samples were fabricated. Here, after a 75.5 nm thick intrinsic Si buffer, Si:C layers with a common thickness of 9 nm (similar to the diode structures described above), but with variable C concentration ranging from 2.2 × 10^17^ cm^–3^ to 5.0 × 10^20^ cm^–3^, were grown at a T_S_ = 200 °C. The C deposition rates were calibrated using secondary-ion mass spectrometry (SIMS) experiments of calibration layers. Finally, the samples were capped with intrinsic Si of 105 nm thickness deposited at T_S_ = 300 °C.

### Device Fabrication

We processed the MBE-grown p-i-n structures (Figure 1) into square mesa diodes with side lengths of 400 μm using standard Si processing techniques in a cleanroom environment. To enable EL emission perpendicular to the diode surface, the electrical metal contact (3 nm of Ti and 400 nm of Au) to the top n-type layer was implemented as a ring and grid contact, see inset of Figure 1(c). As the bottom contact, at the substrate’s backside, the same layer structure of Ti–Au was used, followed by conductive silver paste bonding to a chip carrier. No sidewall passivation was applied. Gold wire bonding was performed to drive the current to the device’s top.

### Spectroscopic Characterization

The CC diode samples were driven by a Keithley 2601-B Pulse source measure unit (SMU). Current–voltage (I–V) curves for a current up to 300 mA were recorded in continuous mode by increasing voltage steps, while for higher currents, the SMU was operated in pulsed mode with 50 μs current pulses to avoid excessive device heating and thermal overloading of the bonding wires. Voltage measurements were done at the device contacts (4-point method) to exclude the voltage drop caused by the connection strings. The EL data was recorded in a sample temperature range from 5 to 100 K, with the emitted light collected by an infinity-corrected 10× Olympus objective with a numerical aperture of 0.26. The collected signal was coupled free-space into a (Teledyne Princeton Instruments) SpectraPro HRS-750 Czerny-Turner type spectrometer with switchable gratings and a connected liquid nitrogen-cooled linear 1024 pixel InGaAs photodiode array with an effective cutoff energy of 0.775 eV (1600 nm). Additionally, we performed microphotoluminescence (μ-PL) measurements at low sample temperatures. For excitation, we used a continuous-wave (cw) diode-pumped solid-state (DPSS) laser emitting at 473 nm and a laser power of max. Six mW (measured below the cryostat window). The laser was focused, and the luminescence signal was collected via an infinity-corrected microscope objective with 0.26 numerical aperture (NA) for the ensemble measurements. The μ-PL spectra were recorded via a 500 mm focal-length Czerny-Turner spectrometer with three interchangeable ruled gratings (100, 300, and 900 l/mm) connected to a liquid-nitrogen-cooled 1024 pixel InGaAs line detector.

## Results and Discussion

Figure 1(b) depicts the sample structure of the SiCCs confined to a 9 nm thick layer and embedded in the ultrathin i-region of the p-i-n junction of the semiconductor diode. Based on previous work,^28^ we have grown the SiCC layer at 200 °C and using a relatively high C-concentration of 3.8 × 10^19^ cm^–3^, for which a strong luminescence response can be obtained (see the Supporting Information (SI)). We note that the resulting SiCC is predominantly not the traditional G-Center that is known to have a zero-phonon line (ZPL) at ∼ 1278 nm. Instead, here we find a derivate of the conventional G-center that we labeled G’-center before.^28^ *Ab initio* calculations of various atomistic configurations strongly indicate that the G’-center consists of a conventional G-center, i.e., two substitutional C atoms combined with an interstitial Si atom (Si_i_), that is accompanied by an additional substitutional C atom, see Figure 1(c).^28^ Such a defect configuration leads to the observed wavelength shift of the ZPL to ∼ 1300 nm while conserving the phonon spectrum properties of the G-center and its favorable spin properties.^28^ Such an atomic configuration is likely since our MBE C-effusion cell is no pure source of atomic C but, in addition, emits molecules with three C atoms that might be built in as a unit into the Si crystal as a consequence of the low T_S_ of 200 °C, facilitating the dominant formation of G’-centers.

We note that the G’-centers offer a higher temperature stability against thermal annealing compared to conventional G-centers. Furthermore, in temperature-dependent PL spectroscopy, higher activation energies for the thermal quenching of the PL is found for G’-centers as compared to G-centers. Our *ab initio* calculations corroborate these findings by comparing the stability and charge transition levels of the two defects. Our results reveal that the activation energy is associated with the thermal ionization from the neutral excited state to the negative charge which also ejects a hole in the process. For a detailed comparison of the optical properties of these emitters, please see the Supporting Information.

In this previous work on self-assembled Si color centers,^28^ which relies on PL studies of the SiCC emission only, we found that an overgrowth temperature of T_S_ = 300 °C provides a good compromise between preservation of the SiCCs, i.e., the absence of significant thermal SiCC annihilation, and an excellent epitaxial quality of the Si capping layer. We note that we intentionally grew the p-type contact at the bottom and the n-type contact at the top of the layer sequence since dopant activation is possible at lower temperatures for Sb than for B.^31,32^ Thus, the higher temperature needed for B-doping activation is employed before the growth of the SiCC layer. Hence, no further annealing steps after the diode growth have been required to activate the dopants, facilitating the preservation of the optical properties of the created SiCCs. Figure 1(d) depicts temperature-dependent I–V curves, showing clear diode characteristics and demonstrating that the low growth temperature used to grow the Si:Sb top contact layer is not detrimental to diode functionality. The insert depicts a photograph of the processed device in which the Ti–Au top-contact grid and the bonding wires are visible.

Figure 2(a) shows EL spectra, demonstrating emission from the SiCCs, confined to the 9 nm thick layer at the p–n junction. The blue spectrum was recorded for an applied excitation current density (I_Appl_) of 625 mA/cm^2^, and the red spectrum for I_Appl_ = 1875 mA/cm^2^. The spectral shape is dominated by the G’ ZPL at a wavelength of 1300.6 nm at 5 K that shifts to 1303 nm at 80 K. A pronounced phonon sideband can be observed at longer wavelengths containing the local phonon mode (LPM) at 1406 nm at 5 K. Water vapor absorption influences the spectral shape in the wavelength range of 1360 to 1420 nm.

**Figure 2:**
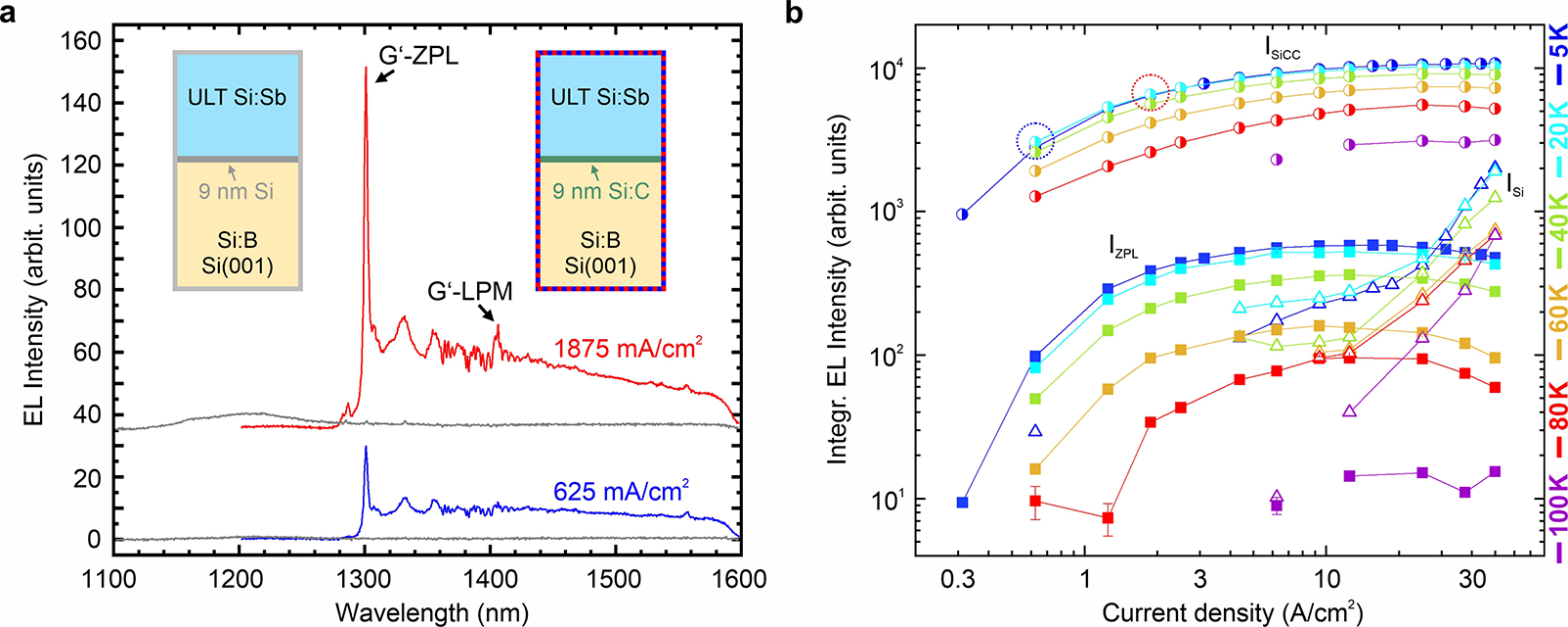
(a) Electroluminescence spectra from a 9 nm thick SiCC layer obtained for applied current densities I_*Appl*_ of 625 mA/cm^2^ (blue) and 1875 mA/cm^2^ (red). Gray spectra show the EL signal from the Si reference diode under the same excitation conditions. Spectra are vertically shifted for clarity. The inserts mark the respective sample layouts. (b) Sample temperature-dependent EL intensity versus current density for the total integrated color center emission I_SiCC_ (half-spheres, 1270 nm -1600 nm), the integrated intensity of the G’ ZPL, I_ZPL_ is indicated by full squares and the intensity from Si bulk by empty triangles. Sample temperatures of 5, 20, 40, 60, 80, and 100 K, are indicated by blue, cyan, green, orange, red, and violet color.

The gray spectra in Figure 2(a) mark the optical response from the pure Si diode obtained at the same applied current densities and for the same geometrical diode design. The insert in Figure 2(a) depicts the sample schemes for the SiCC and Si reference diode, respectively. The clear difference in the integrated intensities indicates the efficient carrier capture and recombination in the SiCCs. Figure 2(b) presents the accumulated results of the integrated and temperature-dependent EL intensities of the SiCC sample versus excitation current density. Data points in blue, cyan, green, orange, red, and violet color correspond to sample temperatures of 5, 20, 40, 60, 80, and 100 K, respectively. The total integrated intensity of the SiCC emission, I_SiCC_, is depicted by the half circles, the fitted integrated intensity of the G’ ZPL is indicated by full squares, and the emission from bulk Si at higher current densities is plotted as open triangles. We note that already for very low current densities >1 A/cm^2^, saturation of the SiCC emission intensity is observed, indicating the low total amount of emission centers in the only 9 nm thick layer.

Figure 3(a) depicts temperature-dependent EL Spectra from the G’-center diode for an excitation current density of 1.88 A/cm^2^ and sample temperatures of 5, 20, 40, 80, and 100 K indicated by blue, cyan, green, orange, red, and violet color, respectively. This thermal quenching behavior of the G’-center EL is similar to that of conventional G-centers and is owed to the defect level structure within the Si band structure. Here, we found an activation energy for thermal quenching of the EL of the G’-center of ∼ 25 meV. Figure 3(b) depicts excitation current density-dependent EL spectra obtained for a sample temperature of 5 K and I_Appl_ ranging from 0.31 A/cm^2^ to 4.38 A/cm^2^.

**Figure 3:**
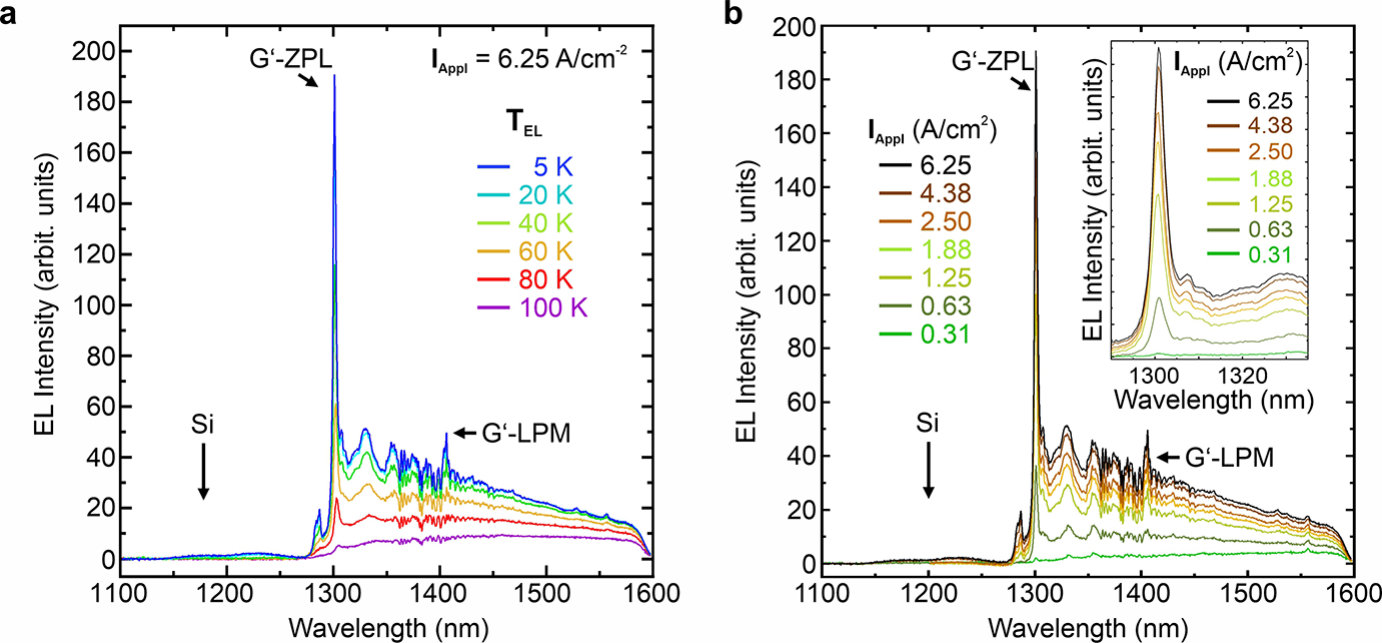
(a) *T*_EL_-dependent EL spectra from G’-centers embedded in a 9 nm thick layer for an applied excitation current density I_Appl_ of 1.88 A/cm^2^. Blue, cyan, green, orange, red, and violet color mark sample temperatures of 5, 20, 40, 60, 80, and 100 K. (b) Current-density-dependent EL spectra at 5 K for I_Appl_ ranging from 0.31 A/cm^2^ to 4.38 A/cm^2^. The inset shows a zoom-in of the I_Appl_-evolution of the G’-center ZPL.

In Figure 4(a), we plotted the integrated intensity of the ZPL of the G’-center emission (I_ZPL_) versus I_Appl_, which yields a superlinear function. Notably, after an initial increase in the fwhm of the G’-ZPL with I_Appl_, the fwhm decreases with increasing I_Appl_ from 3.6 to 3.2 nm, see Figure 4(a).

**Figure 4:**
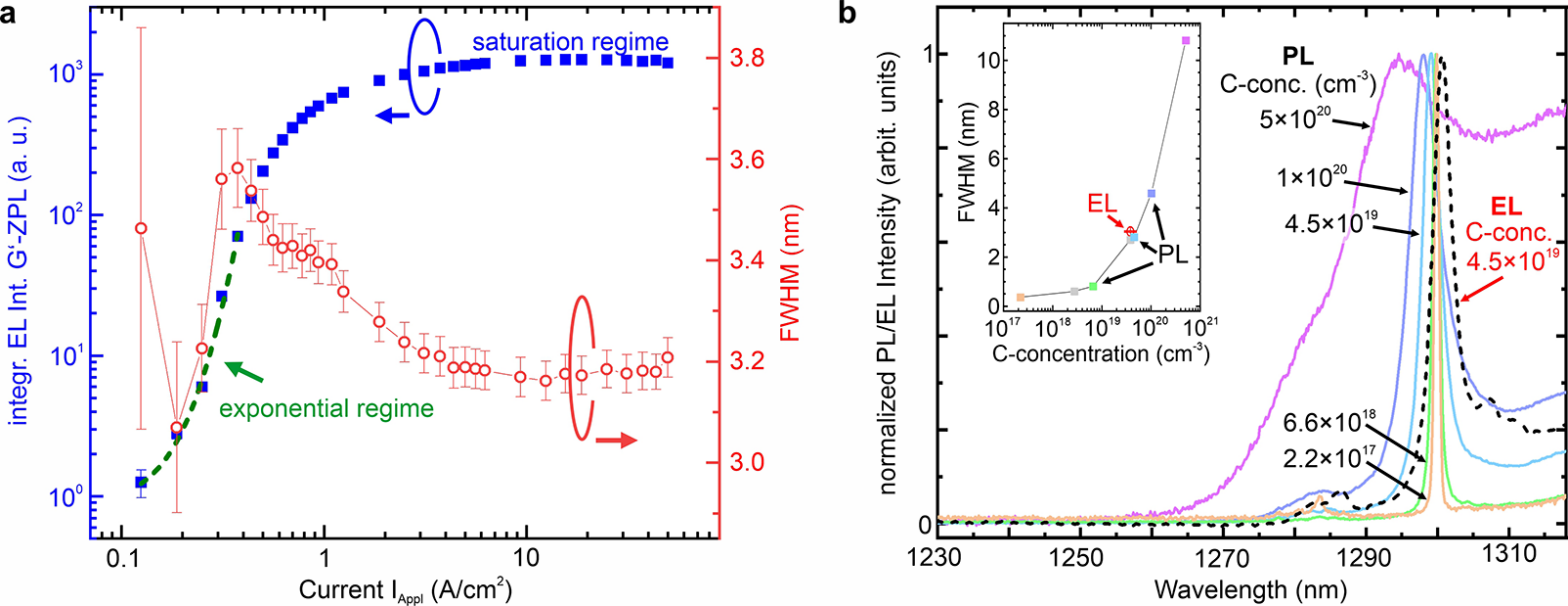
(a) Integrated EL intensity, I_ZPL_ of the G’-centers versus I_Appl_ (blue full squares). The steepest slope shows a clear superlinear (exponential) increase of I_ZPL_ versus I_Appl_, followed by the saturation of I_ZPL_with increasing I_Appl_. The green dashed line shows an exponential fit to the data. The G’-center ZPL fwhm increases in the superlinear regime and decreases toward the saturation regime. (b) Solid spectra: Normalized PL response of G’-centers embedded in 9 nm thick layers and varying C-concentration, 2.2 × 10^17^, 6.6 × 10^18^, 4.5 × 10^19^, 1.0 × 10^20^, and 5.0 × 10^20^ cm^–3^, obtained at 5 K. The dashed black spectrum shows the EL response of the SiCC layer at 5 K and for I_Appl_ = 2.5 A/cm^2^. The inset shows the change of the fwhm of the G’-ZPL with C-content. The red data point indicates that the line width was obtained from EL measurements for I_Appl_ = 2.5 A/cm^2^.

We explain these experimental findings by the nature of the G’-center in Si. In the intrinsic C-doped layer, the G’-center defect is in the optically active neutral state. When this ultrathin intrinsic layer is sandwiched into the p–n junction, many G’-centers will be charged due to the close proximity to the relatively thin depletion layer. By adding and increasing bias voltage into the forward region, the depletion region shrinks, and the quasi-Fermi level shifts toward the midgap at the edges of the ultrathin intrinsic layer. As a consequence, the G’-centers will be mostly neutral. This rise in the concentration of neutralized G’-centers goes exponentially with the applied bias voltage. By assuming that the Auger-ionization process is more efficient for the charged G’-centers than the neutral G’-centers, the carriers can recombine radiatively via the G’-center’s deep states, causing electroluminescence. Thus, the superlinear I_ZPL_ upon increasing bias voltage can be well explained by this effect. Indeed, the exponential function fits well with the observed data points (green dashed curve in Figure 4(a)). The ionization processes result in fluctuating charges around the electroluminescent G’-centers that cause spectral diffusion because of the Stark-shift in the G’-center emission, which finally broadens the observed fwhm in the ZPL emission. By stabilizing the charge state of G’-centers with increasing bias voltage, the concentration of charge fluctuators (charged G’-centers) around the electroluminescent G’-centers decreases, narrowing the fwhm in the ZPL emission of the electroluminescent G’-centers.

Figure 4(b) aims to show that this relatively large fwhm is a direct consequence of the C-concentration used for creating the color centers in the Si:C layer. In the solid lines in Figure 4(b), we compare normalized PL spectra of 9 nm thick SiCC layers grown at 200 °C and overgrown by Si at a temperature of 300 °C, for which different C-doping concentrations between 2.2 × 10^17^ cm^–3^ to 5.0 × 10^20^ cm^–3^ were used to create the G’-centers. For non-normalized PL spectra versus C-concentration, please refer to the SI. With decreasing C-concentration in the nanolayer, a very pronounced line width narrowing of the G’-ZPL is evident from the PL spectral shape (Figure 4(b)). The gray, magenta, dark blue, light blue, green, and orange data points in the inset of Figure 4(b) depict the fitted PL-fwhm of the G’-ZPL versus applied C-doping concentration, showing that the line width decreases from ∼ 10 nm to less than 0.4 nm (∼ 300 μeV) for the lowest C-concentration. The red data point in the inset of Figure 4(b) marks the fwhm obtained from EL (C-concentration of 4.5 × 10^19^ cm^–3^), and the corresponding spectrum is plotted in black in Figure 4(b). Thus, for the same applied C-doping concentration, the line widths obtained from PL and EL are in good agreement, hinting to the influence of the C-concentration on the fwhm of the G-center EL emission. We attribute this behavior to the nonuniform strain field produced by the neutral G’-centers themselves, which vanishes with the lower defect concentration, as observed in PL experiments.^28^

The fabrication of SiCCs without ion implantation is intimately linked to ultralow temperature epitaxy, ULT, as presented here. In previous works, we found that Si, SiGe, and Ge layers, grown at ULT < 350 °C, can be grown with high epitaxial quality if the chamber pressure during the growth is sufficiently low (≲ 3 × 10^–10^ mbar). In contrast to the growth at conventional, high temperatures >500 °C, where the sticking coefficients of residual gas atoms on the substrate are small, at temperatures <350 °C sticking coefficients become large, and atoms cannot be desorbed from the substrate surface,^33^ leading to their incorporation into the crystal. Figure S1 in the SI presents the excellent maximum growth pressure of 3 × 10^–10^ mbar, enabling good crystal quality despite low growth temperature. Such growth conditions also enable novel device schemes based on previously unattainable layer stacks of defect-free and fully strained SiGe and Ge heterostructure layers directly grown on Si or SOI.^34–36^ Furthermore, type-I double heterostructure diodes can be achieved based on thick and Ge-rich SiGe/Si(001) layers.^37^

Here, Si epitaxy at untypically low growth temperatures of 200 °C concomitant with carbon codoping enables a balance between crystalline quality, as seen from transmission electron microscopy,^28^ and kinetically induced color center formation leading to the described G’-centers.^28^ For the latter, the growth temperature needs to be low enough to ensure that the movement of the SiCC’s C atoms is kinetically limited to avoid SiCC dissociation. The necessary three C atoms for the G’ formation is provided by the dominant C_3_-emission of the C-sublimation source.^28^

The results demonstrate that an alternative fabrication path for SiCCs in diodes is feasible, notably without ion implantation. The pure epitaxial nature of this fabrication scheme allows for the confinement of the SiCCs in layers of arbitrary thickness and at arbitrary vertical positions in the diode with nanometer resolution.

While the results of this work are a proof-of-concept structure, we note that for lasing applications, a large gain material volume, i.e., a high number of color centers, can be achieved through large layer thicknesses of tens to hundreds of nanometers, and high C-doping concentrations. Theoretical insights will be needed to optimize device and layer designs to find an optimum regarding, e.g., gain material volume, thickness of the intrinsic layer of the p-i-n diode, and doping concentrations.

Additionally, we can envision the implementation of the self-assembled SiCCs into SiGe type-I double heterostructures that can result in an efficient carrier capture in the SiCC recombination region and limit minority carrier injection in the diodes.^37^ Both, thick and fully strained SiGe layers for double heterostructure formation, and SiCC layers rely on epitaxy temperatures <300 °C. Thus, combining these two approaches can be considered to be possible without thermal deactivation and annihilation of the SiCCs. We note that silicon-on-insulator substrates (SOI) can also be employed for the presented SiCC-diode growth scheme. This is particularly important for fabricating photonic resonators toward electrically driven lasers and implementing isolated SiCCs for integrated quantum optics purposes.

While we have used a rather high C-concentration in this work to demonstrate the feasibility of the approach, we emphasize that SiCC self-assembly also opens the path to lower the SiCC density down to isolated emitter levels by either lowering the C-doping concentration, the layer thickness, or a combination of both accompanied by careful thermal annealing at appropriate temperatures. For quantum photonics applications, this all-MBE approach allows for embedding the SiCC quantum emitters in a matrix composed of isotopically purified Si^28^.

## Conclusions

Color center-based LEDs suffer from a wide vertical spread of emitter position if the emitters are formed using ion implantation. This will ultimately limit, e.g., the usability of heterostructures to boost emitter properties. Here, we propose an alternative fabrication scheme for Si color center diodes using ultralow temperature epitaxy and demonstrate that G’-centers, derivates of conventional G-center, can be embedded in an all epitaxial approach into a less than 10 nm thick layer that is deterministically grown at the p–n junction of the LED. Thereby, the emitters sustain the thermal budget needed for top contact formation and dopant activation while the diodes do not suffer from the low-temperature epitaxy. These results pave the way for applications of SiCCs as light emitters in Si photonics and single photon sources that can be manipulated through the electrical fields of the diode.

## Supplementary Material



## Notes

Growth protocol for the epitaxial formation of a SiCC diode, emphasis on the importance of the growth pressure <3 × 10^–10^ mbar for forming Si color centers, growth rates and temperatures, influence of the C-concentration on the PL response of self-assembled Si color centers, influence of the thickness of C-doped layers on the PL response of G′ centers, photoluminescence lifetimes versus C-concentration in G′-centers, comparison of thermal deactivation of G-centers and G′ centers through thermal annealing, lifetime of thermally annealed G′ center ensembles, PL power dependence of G′ center emission, activation energies for charge carrier escape from G′-centers and G-centers, *ab initio* calculations comparing the G and G′ center (PDF)

## References

[1] Davies G. (1989). The optical
properties of luminescence centres in silicon. Phys. Rep..

[2] Udvarhelyi P., Somogyi B., Thiering G., Gali A. (2021). Identification of a
Telecom Wavelength Single Photon Emitter in Silicon. Phys. Rev. Lett..

[3] Cloutier S. G., Kossyrev P. A., Xu J. (2005). Optical gain
and stimulated emission
in periodic nanopatterned crystalline silicon. Nat. Mater..

[4] Berhanuddin D. D., Lourenço M. A., Gwilliam R. M., Homewood K. P. (2012). Co-Implantation
of Carbon and Protons: An Integrated Silicon Device Technology Compatible
Method to Generate the Lasing G-Center. Adv.
Funct. Mater..

[5] Thomson D., Zilkie A., Bowers J. E., Komljenovic T., Reed G. T., Vivien L., Marris-Morini D., Cassan E., Virot L., Fédéli J.-M., Hartmann J.-M., Schmid J. H., Xu D.-X., Boeuf F., O’Brien P., Mashanovich G. Z., Nedeljkovic M. (2016). Roadmap on
silicon photonics. Journal of Optics.

[6] Pavesi L., Lockwood D. J. (2016). Silicon photonics
III. Topics
Appl. Phys..

[7] Shi B., Zhu S., Li Q., Tang C. W., Wan Y., Hu E. L., Lau K. M. (2017). 1.55 μm
room-temperature lasing from subwavelength
quantum-dot microdisks directly grown on (001) Si. Appl. Phys. Lett..

[8] Wan Y., Norman J., Li Q., Kennedy M. J., Liang D., Zhang C., Huang D., Zhang Z., Liu A. Y., Torres A., Jung D., Gossard A. C., Hu E. L., Lau K. M., Bowers J. E. (2017). 1.3 μm
submilliamp threshold
quantum dot micro-lasers on Si. Optica.

[9] Chen S., Li W., Wu J., Jiang Q., Tang M., Shutts S., Elliott S. N., Sobiesierski A., Seeds A. J., Ross I., Smowton P. M., Liu H. (2016). Electrically pumped continuous-wave
III–V quantum dot lasers on silicon. Nat. Photonics.

[10] Knoch J., Richstein B., Han Y., Jungemann C., Icking E., Schreiber L. R., Xue R., Tu J.-S., Gökcel T., Neugebauer J., Stampfer C., Zhao Q. T. (2023). On the
Performance of Low Power Cryogenic Electronics for Scalable Quantum
Information Processors. IEEE Nanotechnology
Materials and Devices Conference (NMDC).

[11] Redjem W., Durand A., Herzig T., Benali A., Pezzagna S., Meijer J., Kuznetsov A. Y., Nguyen H. S., Cueff S., Gérard J. M., Robert-Philip I., Gil B., Caliste D., Pochet P., Abbarchi M., Jacques V., Dréau A., Cassabois G. (2020). Single artificial atoms in silicon emitting at telecom
wavelengths. Nature Electronics.

[12] Hollenbach M., Berencén Y., Kentsch U., Helm M., Astakhov G. V. (2020). Engineering
telecom single-photon emitters in silicon for scalable quantum photonics. Opt. Express.

[13] Baron Y., Durand A., Udvarhelyi P., Herzig T., Khoury M., Pezzagna S., Meijer J., Robert-Philip I., Abbarchi M., Hartmann J.-M., Mazzocchi V., Gérard J.-M., Gali A., Jacques V., Cassabois G., Dréau A. (2022). Detection of single W-centers in silicon. ACS Photonics.

[14] Higginbottom D. B., Kurkjian A. T. K., Chartrand C., Kazemi M., Brunelle N. A., MacQuarrie E. R., Klein J. R., Lee-Hone N. R., Stacho J., Ruether M., Bowness C., Bergeron L., DeAbreu A., Harrigan S. R., Kanaganayagam J., Marsden D. W., Richards T. S., Stott L. A., Roorda S., Morse K. J., Thewalt M. L. W., Simmons S. (2022). Optical observation
of single spins in silicon. Nature.

[15] Bergeron L., Chartrand C., Kurkjian A. T. K., Morse K. J., Riemann H., Abrosimov N., Becker P., Pohl H.-J., Thewalt M. L. W., Simmons S. (2020). Silicon-Integrated Telecommunications Photon-Spin Interface. *PRX*. Quantum.

[16] Lee K. M., O'Donnell K. P., Weber J., Cavenett B. C., Watkins G. D. (1982). Optical
detection of magnetic resonance for a deep-level defect in silicon. Phys. Rev. Lett..

[17] Krithika, V. R. , Coherent spin control of telecom single-photon emitters in silicon; Presenter: Félix Cache. APS March meeting: https://summit.aps.org/events/MAR-X19/1: (accessed 2025–04–13).

[18] Day A. M., Sutula M., Dietz J. R., Raun A., Sukachev D. D., Bhaskar M. K., Hu E. L. (2024). Electrical manipulation of telecom
color centers in silicon. Nat. Commun..

[19] Buckley S. M., Tait A. N., Moody G., Primavera B., Olson S., Herman J., Silverman K. L., Papa Rao S., Woo Nam S., Mirin R. P., Shainline J. M. (2020). Optimization
of photoluminescence from W centers in silicon-on-insulator. Opt. Express.

[20] Bao J., Tabbal M., Kim T., Charnvanichborikarn S., Williams J. S., Aziz M. J., Capasso F. (2007). Point defect
engineered
Si sub-bandgap light-emitting diode. Opt. Express.

[21] Murata K., Yasutake Y., Nittoh K. i., Fukatsu S., Miki K. (2011). High-density
G-centers, light-emitting point defects in silicon crystal. AIP Adv..

[22] Canham L. T., Barraclough K. G., Robbins D. J. (1987). 1.3-μm light-emitting diode
from silicon electron irradiated at its damage threshold. Appl. Phys. Lett..

[23] Rotem E., Shainline J. M., Xu J. M. (2007). Electroluminescence of nanopatterned
silicon with carbon implantation and solid phase epitaxial regrowth. Opt. Express.

[24] Ebadollahi N., Namboodiri P. N., Pederson C., Veetil V. K., Davanco M. I., Srinivasan K. A., Katzenmeyer A. M., Pelton M., Pomeroy J. M. (2024). Fabrication
of silicon W and G center embedded light-emitting diodes for electroluminescence. J. Vac. Sci. Technol. B.

[25] Day A. M., Zhang C., Jin C., Song H., Sutul M., Sipahigi A., Bhaskar M. K. (2025). Probing negative differential
resistance in silicon with a PIN diode-integrated T center ensemble. arXiv preprint.

[26] Anderson C. P., Bourassa A., Miao K. C., Wolfowicz G., Mintun P. J., Crook A. L., Abe H., Ul Hassan J., Son N. T., Ohshima T., Awschalom D. D. (2019). Electrical
and optical control of single spins integrated in scalable semiconductor
devices. Science.

[27] Ziegler J. F., Ziegler M. D., Biersack J. P. (2010). SRIM–The stopping and range
of ions in matter (2010). Nuclear Instruments
and Methods in Physics Research Section B: Beam Interactions with
Materials and Atoms.

[28] Aberl J., Navarrete E. P., Karaman M., Enriquez D. H., Wilflingseder C., Salomon A., Primetzhofer D., Schubert M. A., Capellini G., Fromherz T., Deák P., Udvarhelyi P., Li S., Gali Á., Brehm M. (2024). All-Epitaxial
Self-Assembly of Silicon
Color Centers Confined Within Sub-Nanometer Thin Layers Using Ultra-Low
Temperature Epitaxy. Adv. Mater..

[29] Liu J., Konthasinghe K., Davanço M., Lawall J., Anant V., Verma V., Mirin R., Nam S. W., Song J. D., Ma B., Chen Z. S., Ni H. Q., Niu Z. C., Srinivasan K. (2018). Single Self-Assembled
InAs/GaAs Quantum Dots in Photonic Nanostructures: The Role of Nanofabrication. Physical Rev. Appl..

[30] Salomon A., Aberl J., Vukušić L., Hauser M., Fromherz T., Brehm M. (2022). Relaxation delay of
Ge-rich epitaxial
SiGe films on Si (001). Phys. Status Solid.
A.

[31] Eberl K., Iyer S. S., Delage S. L., Ek B. A., Cotte J. M. (1991). Boron Doping
in Si-MBE. MRS Proceedings.

[32] Gossmann H.-J., Schubert E. F., Eaglesham D. J., Cerullo M. (1990). Low-temperature Si
molecular beam epitaxy: Solution to the doping problem. Appl. Phys. Lett..

[33] Yabumoto, N. Analysis of molecular adsorbates on Si surfaces with thermal desorption spectroscopy. AIP Conf. Proc.; American Institute of Physics 1998, 449, 696-701.

[34] Wind L., Sistani M., Böckle R., Smoliner J., Vukŭsić L., Aberl J., Brehm M., Schweizer P., Maeder X., Michler J., Fournel F., Hartmann J., Weber W. M. (2022). Composition Dependent Electrical Transport in Si_1–x_Ge_x_ Nanosheets with Monolithic Single-Elementary
Al Contacts. Small.

[35] Wilflingseder C., Aberl J., Prado Navarrete E., Hesser G., Groiss H., Liedke M. O., Butterling M., Wagner A., Hirschmann E., Corley-Wiciak C., Zoellner M. H., Capellini G., Fromherz T., Brehm M. (2024). Ge Epitaxy
at Ultralow Growth Temperatures
Enabled by a Pristine Growth Environment. ACS
Appl. Electron. Mater..

[36] Fuchsberger A., Wind L., Sistani M., Behrle R., Nazzari D., Aberl J., Prado Navarrete E., Vukŭsić L., Brehm M., Schweizer P., Vogl L., Maeder X., Weber W. M. (2023). Reconfigurable Field-Effect Transistor Technology via
Heterogeneous Integration of SiGe with Crystalline Al Contacts. Adv. Electron. Mater..

[37] Salomon A., Aberl J., Vukušić L., Navarrete E. P., Marböck J., Enriquez D.-H., Schuster J. (2024). A group-IV
double heterostructure light emitting diode for room temperature gain
in Silicon. arXiv.

