# Supplementary material for: Telecom Light-Emitting Diodes Based on Nanoconfined Self-Assembled Silicon-Based Color Centers
Source: ACS Photonics. 2025 May 7;12(5):2364–71. doi: 10.1021/acsphotonics.4c01662 (PMC12100701; doi:10.1021/acsphotonics.4c01662)
Supplement: Supplementary file 1 [file ph4c01662_si_001.pdf]

Supporting Information

Telecom light-emitting diodes based on nanoconfined self-assembled silicon-based color centers

Andreas Salomon[a], Johannes Aberl[a], Enrique Prado Navarrete[a], Merve Karaman[a], Oliver E. Lang[a], Daniel Primetzhofer[b], Peter Deák[c,d], Ádám Gali[c,e,f], Thomas Fromherz[a], Moritz Brehm[a]*

[a] *Institute of Semiconductor and Solid State Physics, Johannes Kepler University, Altenberger Straße 69, Linz 4040, Austria*

[b] *Department of Physics and Astronomy, Uppsala University, Box 516, 75120 Uppsala, Sweden*

[c] *HUN-REN Wigner Research Centre for Physics, P.O. Box 49, H-1525, Budapest, Hungary*

[d] *Beijing Computational Science Research Center, Beijing 100193, China*

[e] *Department of Atomic Physics, Institute of Physics, Budapest University of Technology and Economics, Műegyetem rakpart 3., Budapest H-1111, Hungary*

[f] *MTA-WFK Lendület "Momentum" Semiconductor Nanostructures Research Group, P.O. Box 49, H-1525, Budapest, Hungary*

* Email: moritz.brehm@jku.at

13 pages, 10 figures, 1 table

Growth parameters during molecular beam epitaxy

We argue that an excellent growth pressure is essential for ultra-low temperature growth. Impurity atoms that contribute to the background pressure and that are impinging on the sample surface during growth cannot be efficiently desorbed from the surface at temperatures lower than ~350°C (1). Their incorporation into the crystal lattice leads to the formation of unwanted point defects and, eventually, at larger layer thicknesses, the breakdown of the epitaxial growth. Therefore, lower growth pressures during deposition significantly improve the layers' crystalline quality for ULT growth (2-5). Note that the growth pressure is not significantly worse for the diode growth as compared to intrinsic layers for photoluminescence investigations (6), despite the high temperatures of the effusion cells employed.

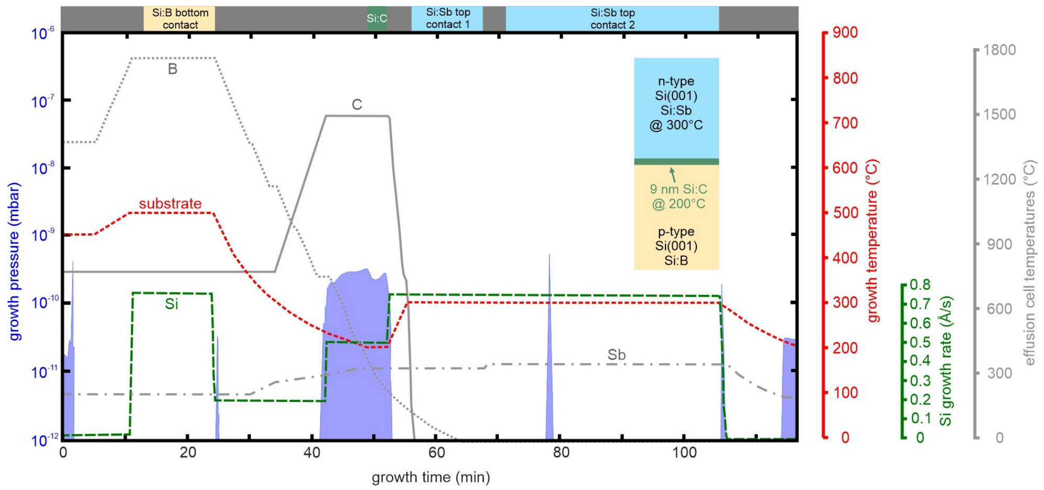

Figure S1: *Growth protocol of the LED containing self-assembled SiCCs. Very low growth pressures enable excellent layer qualities even for ultra-low-temperature growth. Growth pressure (left ordinate) is indicated by the blue-shaded areas. The right ordinates correlate to the substrate temperature (red dotted line), Si growth rate (green dashed line), and effusion cell temperatures for the carbon- (solid grey line), boron- (grey dotted line), and antimony source (grey dashed-dotted line). Within the grey-shaded areas, the growth temperature or the emission rates were ramped to their respective setpoints with all effusion cells and evaporators closed. The inset depicts the sample structure.*

Photoluminescence emission intensities of self-assembled carbon-based Si color centers

Figures S2 and S3 present the PL intensities in counts per second of epitaxially grown G' centers versus the used C-doping concentration (Fig. S2) and versus Si:C layer thickness. As mentioned in the experimental methods section of the main text, the PL emission was detected using a conventional Pylon InGaAs line detector, i.e., not a single photon detector. In Figure S2, the PL response is plotted for a 9 nm thick Si:C layer that was doped with 6.6×10^{18} cm^{-3}, 3.8×10^{19} cm^{-3}, 4.5×10^{19} cm^{-3}, 1.0×10^{20} cm^{-3}, and 5.0×10^{20} cm^{-3}, respectively. We observe an increase in the peak PL intensity with increasing C-concentration up to 4.5×10^{19} cm^{-3}. That is, however, accompanied by a significant linewidth broadening of the G'-ZPL line, see Fig. S2(b) and Ref. (7).

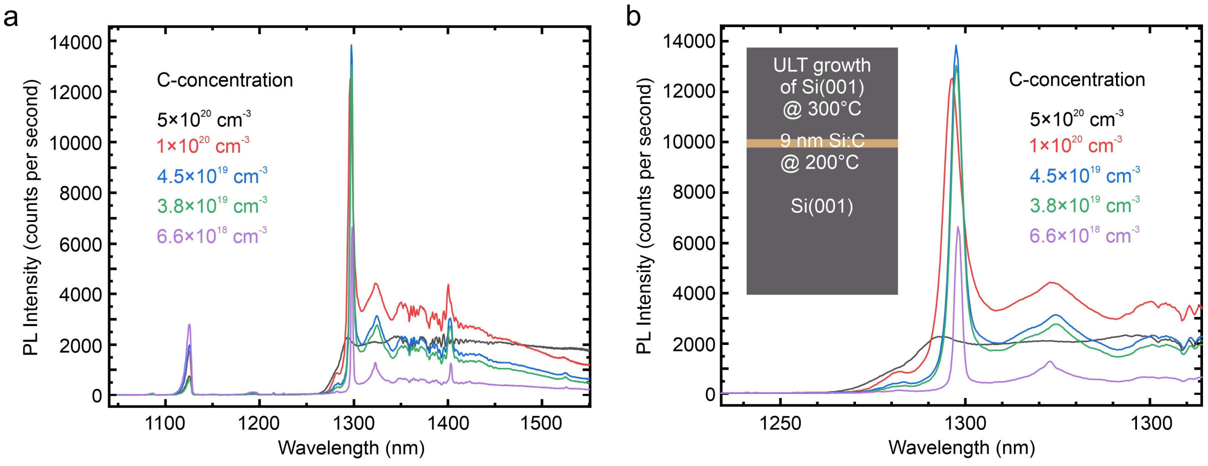

Figure S2: *Photoluminescence spectra of G' centers created epitaxially within a 9 nm thick layer C-doped Si layer, grown at 200°C for various C-doping concentrations. (a) shows the whole spectrum, including the phonon-side band, the local phonon mode and the Si bulk peak with intensities given in counts per second. (b) shows the zoom-in on the ZPL, indicating a pronounced linewidth narrowing for smaller implantation dose. The inset in (b) indicates the sample layout for the PL experiments.*

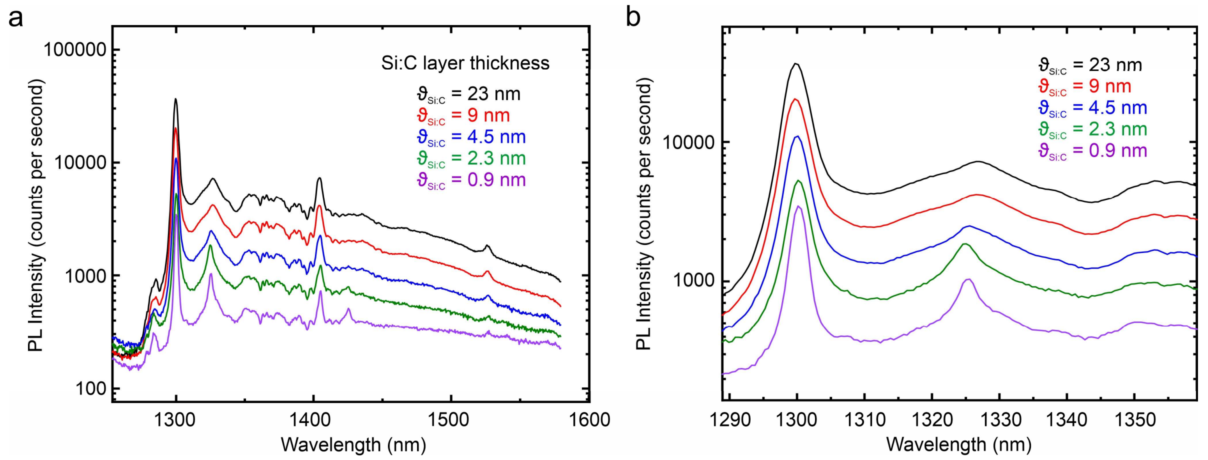

Figure S3: *Photoluminescence spectra of G' centers created epitaxially within C-doped Si layer ($C = 3.8\times10^{19}$ cm^{-3}) of different thickness $\vartheta_{Si:C}$, grown at 200°C and overgrown with Si at 300°C. (a) Whole spectrum, including the phonon-side band, the local phonon mode and the Si bulk peak with intensities given in counts per second. (b) shows the zoom-in on the ZPL, indicating only a light influence of the FWHM with a changing layer thickness.*

A straightforward way to increase the emission intensity of self-assembled epitaxial G'-centers while keeping variations in the FWHM of the ZPL low is to increase the thickness of the Si:C doped layer. Figure S3 shows the PL response for Si:C layers grown at 200°C with a doping concentration of 3.8×10^{19} cm^{-3} and a varied layer thickness from less than 1 nm to 23 nm. The PL response scales with the layer thickness and peak count rates of ~40000 counts per second were recorded for the thickest layer. We note that there is no intrinsic limit to the layer thickness.

Time-resolved PL of self-assembled carbon-based Si color centers for different C-concentrations

To elucidate the carrier recombination behavior of G'-centers formed under varying carbon doping concentrations, we performed time-resolved photoluminescence (PL) measurements. The same samples as described in the previous section were utilized. For these measurements, a bandpass filter at 1300 nm with a full width at half maximum (FWHM) of 12 nm was employed to select the time-resolved PL of the zero-phonon line (ZPL) of the G'-centers. Single nanowire single-photon detectors (SNSPDs) from Single Quantum, with detection efficiencies of more than 80% at the wavelength of 1300 nm, were used as detectors. For the sample excitation, a red laser with a laser power of 48.7 μW and a repetition rate of 10 MHz was used.

Figure S4 presents two representative PL time decay spectra from G'-centers formed under a C-doping concentration of 3.8×10^{19} cm^{-3} and 2.2×10^{17} cm^{-3}, resulting in PL lifetimes of 10.1 ns for the higher carbon concentration and 14.1 ns for the lower concentration.

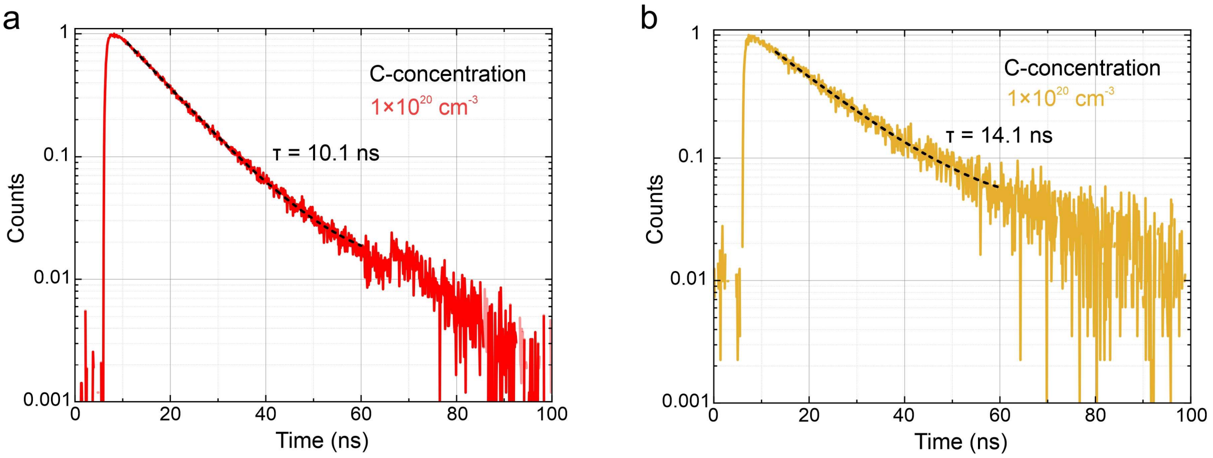

Figure S4: *Time-resolved photoluminescence (PL) decay spectra of G'-centers formed under different carbon doping concentrations. The spectra correspond to carbon concentrations of (a) C =*3.8×10^{19} *cm*$^{-3}$ *(red curve) and (b) C =*2.2×10^{17} *cm*$^{-3}$ *(orange curve). Black dashed lines are single exponential fits to the data. The PL lifetimes are 10.1 ns for the higher carbon concentration and 14.1 ns for the lower concentration.*

Figure S5 shows the fitted lifetimes for all investigated C-concentrations, and a general trend can be seen. As the C-concentration is lowered, the lifetime is increased from about 9 ns at C-concentrations >3.8×10^{19} cm^{-3} to about 14 ns for 2.2×10^{17} cm^{-3}. For conventional G-centers, it was found that isolated emitters have longer lifetimes than ensembles (8).

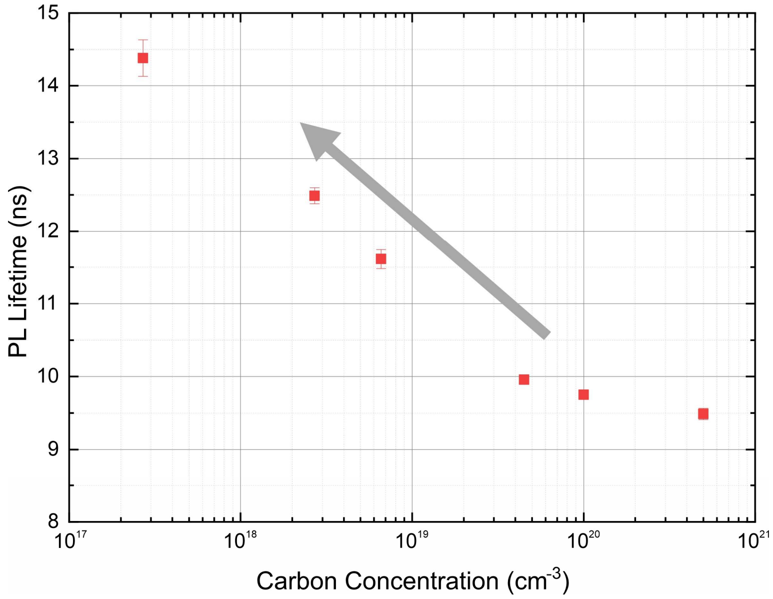

Figure S5: *Summary of the photoluminescence (PL) lifetimes of self-assembled G' centers for different carbon concentrations in the 9 nm thick silicon layer, extracted from time-resolved PL measurements.*

Characteristics of the G'-center with respect to the conventional G-center

In the following section, we aim to deepen the comparison of the optical properties between the G' centers investigated here and the conventional G-centers that can form upon ion implantation into Si. For this purpose, a 300 nm thick Si epilayer was grown on a full 4 inch FZ Si(001) wafer using a Si growth rate of 0.9 Å/s. During the growth of the first 40 nm, the growth temperature (T_G) was linearly ramped from 475°C to 700°C, while the remaining 260 nm were grown at a constant T_G = 700°C. After growth, the wafer was annealed in situ at 750°C for 1 hour. To create high-density common G-centers, the wafer was irradiated with $5 \cdot 10^{14}$ cm^{-2} $^{12}C^{+}$ ions under a tilt angle of 7° using an ion energy of 34.5 keV, which should lead to a mean projected ion range of ~110 nm (7). The ion implantation was performed at room temperature without any post-implantation annealing. For the comparative PL measurements, 4 x 4 mm pieces were cut from the wafer center, followed by a pre-cleaning procedure described previously (7).

For the G-centers formed through carbon ion implantation, we observe the typical spectral fingerprint of the "traditional" G-center ensemble (9), i.e. a bright zero-phonon line (ZPL) at ~1278.5 nm with an ensemble linewidth of 0.57 nm at FWHM, an extended phonon side band with a local phonon mode(s) (LPM, also called E-line) at ~1381 nm (and ~1500.8 nm) and a Debye-Waller factor of ~17% at 5 K. PL spectra comparing the G-center and the G'-center can be found in the supplementary material of Ref. (7).

In the following, we will describe the stability of G and G' centers against thermal annealing, the influence of thermal annealing on the PL decay properties of G' centers, PL quenching and activation

energies for G and G’ centers, and the PL power dependence and PL intensity saturation behavior for G-centers and G’ centers.

- **Stability of the G’ center against thermal annealing**

We cut a G center sample and a G’ center sample into 4×4mm^2 pieces to perform thermal annealing at different temperatures for identical samples. Annealing was performed under a nitrogen atmosphere in an annealing oven ULVAC Riko Mila 5000 for 20 min each. The annealing temperature was varied from 275°C to 400°C and the results of the integrated PL intensities of the G- and G’ centers are presented in Fig. S6. The PL intensity values were normalized to the respective integrated intensities before thermal annealing. As expected from previous results from Davies et al. (9), annealing of the G center at temperatures above 250°C leads to pronounced quenching of the PL intensity. Here, the fit of the decreasing PL intensities with increasing annealing temperatures results in an activation energy for color center dissociation of E_A = 1.9 eV for G-centers. For self-assembled G’-centers, the activation energy is significantly higher with a fitted value of 2.6 eV. This result implies that the strain field generated by the complex is less developed by the G’-centers which might be explained by replacing silicon atom by a carbon atom near the G-center, as the interstitial atom in the G-center introduces a compressive strain field to the lattice, whereas a nearby substitutional carbon atom introduces a tensile strain field. Further discussion can be found in the *ab initio* modeling section below.

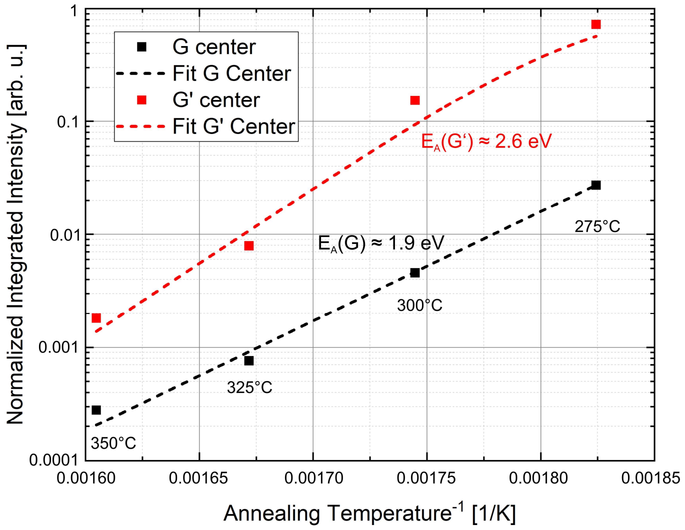

Figure S6: *Summary of the integrated PL-emission intensity of self-assembled G’ centers for different C-concentration in the 9 nm thick Si layer. Integrated PL intensities are normalized to the respective integrated PL intensities before thermal annealing. Black squares (red squares): PL intensity of G-centers (G’-centers) after a 20 min annealing at the temperatures of 275°C, 300°C, 325°C and 350°C. Dashed curves are fits for the deactivation energies of the respective color centers.*

Silicon-based color centers are generally fragile when subjected to thermal annealing. For device fabrication, such as the LED presented in the main part of this work, an increased thermal budget is certainly advantageous, as processes like metallization and surface passivation require specific thermal budgets. In this context, the increased thermal budget of the G'-centers compared to the conventional G-centers can be seen as a distinct advantage.

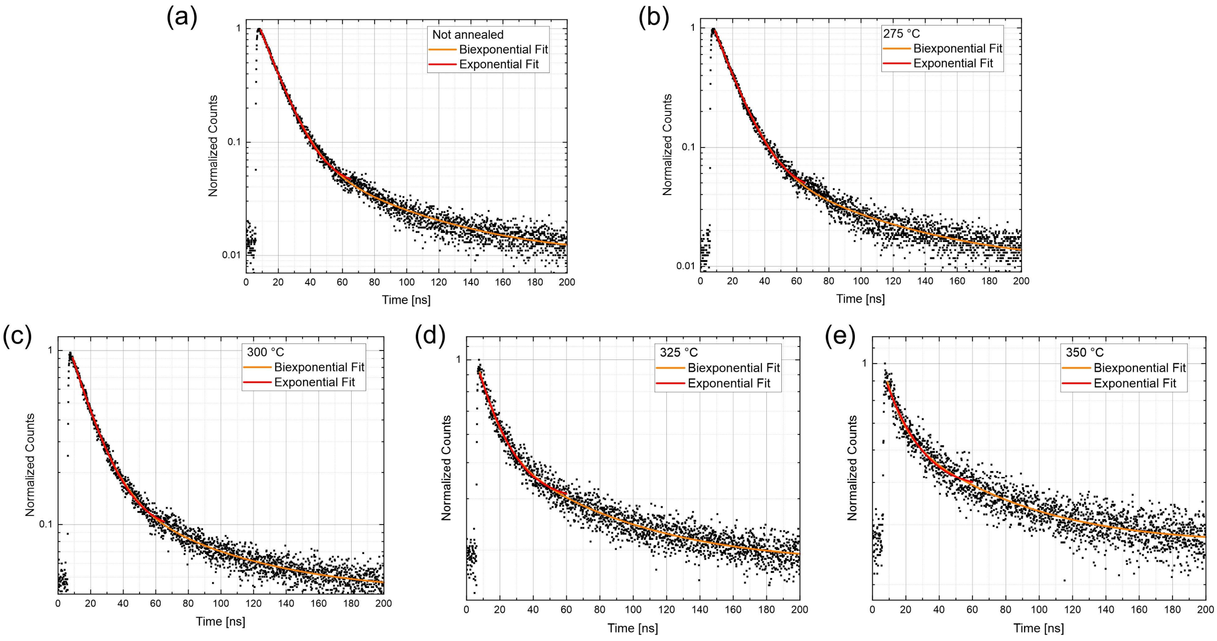

Figure S7: *Photoluminescence (PL) decay curves of self-assembled G' centers with a carbon concentration of* 6.6×10^{18} *cm*$^{-3}$*. (a) Before annealing, and (b)-(e) after annealing for 20 minutes at temperatures of 275°C, 300°C, 325°C, and 350°C. Red and orange curves represent single- and biexponential fits to the PL decay, respectively.*

Figure S7 shows photoluminescence (PL) decay curves of a self-assembled G'-center sample (9 nm thick active layer with a carbon concentration of 6.6×10^{18} cm^{-3} before annealing and after annealing for 20 minutes in a nitrogen atmosphere at temperatures of 275°C, 300°C, 325°C, and 350°C. For the sample excitation, a red laser with a laser power of 21.3 μW and a repetition rate of 5 MHz was used. Red and orange curves represent single-exponential and double-exponential fits to the data, respectively. The extracted PL lifetimes for the differently annealed samples are presented in Table S1. Surprisingly, no significant influence of annealing on the PL lifetime was observed. This finding contrasts with the results shown in Figure S5, where a pronounced increase in lifetime with decreasing carbon content was found. In our recent work, we have shown that the PL-intensity scales with the C-concentration in the film. Therefore, we argue that a lower PL intensity at a lower supplied C-concentration is a consequence of a lower color center density. In this annealing study, we find (see Fig. S6), that the PL intensity of the G'-

centers is decreasing with increasing annealing temperature, suggesting that the number of optically active color centers is decreasing.

Since the lifetime in the annealing study (see Tab. 1) is not increasing with lowering the color center density (as also found in G-centers, Ref (8)), we attribute the low lifetime for the annealed samples to the presence of carbon in optically inactive configurations nearby the remaining G' centers. This further indicates the sensitivity of the color centers to surrounding impurities and imperfections, highlighting the importance of an ultra-clean matrix environment around the emitters.

Table S1: *Summary of the fitted lifetimes of a G'-center sample with 9 nm thick active layer containing a C-concentration of* 6.6×10^{18} cm^{-3} *and a laser (λ=658 nm) excitation power of 21.3 µW and a repetition rate of 5 MHz.*

annealing	lifetime
no	11 ns
275°C	11,6 ns
300°C	12,4 ns
325 °C	11 ns
350°C	9 ns

- **Power-dependence of PL emission of G'-centers**

We performed a series of measurements varying the laser excitation power to investigate the power dependence of the photoluminescence (PL) emission of G'-centers and G-centers. The samples were excited using a blue laser (473 nm) with adjustable power settings ranging from ~0.01 mW to ~3 mW. The PL intensity was recorded for each power setting to determine the relationship between excitation power and PL emission.

In Figure S8, we compare the PL response of implanted G-centers with increasing PL excitation power to epitaxial G'-centers, obtained after co-doping the 9 nm thick Si layer grown at 200°C with C-concentrations of 3.8×10^{19} cm^{-3} and 2.2×10^{18} cm^{-3}. We observe a strictly linear relationship between PL intensity and excitation power in the low-power regime for all samples. The sublinear increase, a sign of charge carrier saturation, is first reached for the low C-concentration sample, suggesting that the color center density is correlated to the C-co-doping concentration. For the implanted G-centers, no saturation of the PL is observed in the investigated range of PL excitation powers. This lack of saturation is attributed to the high carbon dose during implantation of $5\cdot10^{14}$ cm^{-2}. Dashed lines in Fig. S8 are fits to the data according to Ref. 10:

$$I_{PL} = I_0 \cdot \ln\left(1 + \frac{P}{P_{sat}/\ln 2}\right)$$

where P_{sat} is the excitation power at saturation and I_0 is the color center emission intensity for the incident power of $P_{sat} \cdot (e-1)/\ln 2$ (10). From these fits, we can obtain saturation power levels of 15,28W, ~740 mW, and ~58 mW for the Implanted G-center sample and the high and low-concentration G'-center sample, respectively. This finding indicates that the G'-center density is proportional to the supplied C-concentration, as the ratio of the carbon concentration over P_{sat} is the same for both concentrations.

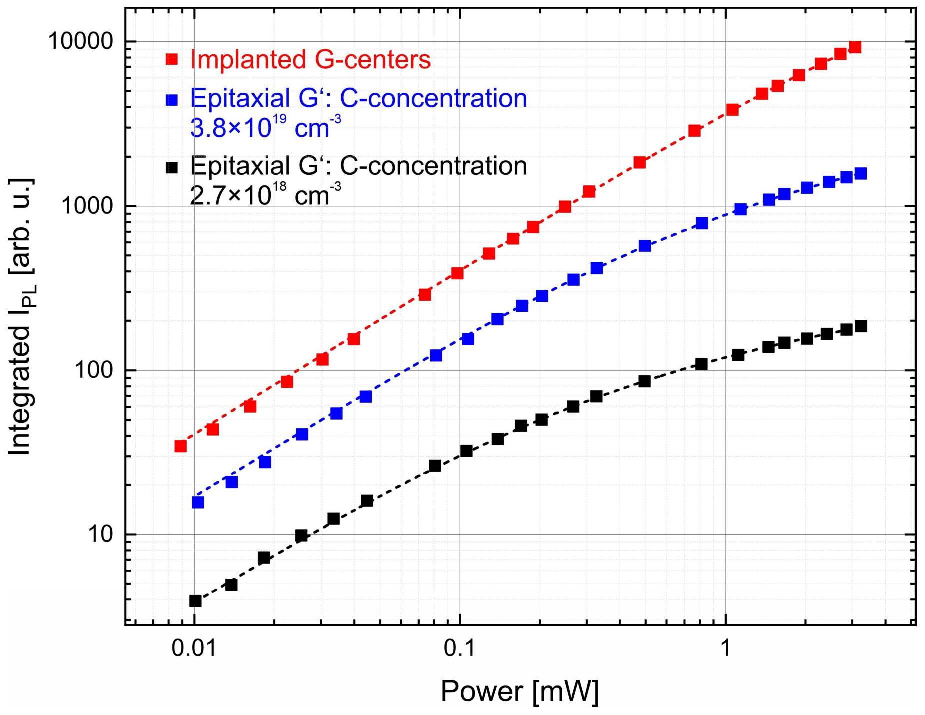

Figure S8: *Integrated PL intensity versus PL excitation power for implanted G-centers and self-assembled G' centers obtained from two different C-concentrations: 3.8×10^{19} cm^{-3} and 2.2×10^{18} cm^{-3}. Dashed curves are fits to the data.*

In Fig. S9, we investigate the influence of the sample temperature on the PL emission intensity. From these Arrhenius plots, activation energies can be determined for the thermal escape of the charge carriers from the color centers. The activation energies for thermal PL quenching from Arrhenius plots are calculated according to $I(T) = I_0 \cdot \left(1 + A \cdot \exp\left(\frac{-E_A}{k_B T}\right)\right)^{-1}$, where I(T) is the temperature-dependent integrated intensity, I_0 is the intensity at 0 K, E_A is the activation energy, k_B is the Boltzmann constant, T is the temperature, and A is a fitting parameter.

An activation energy of about 40 meV was found for the ensemble of conventional G-centers, in agreement with previously published work in G-center ensembles (10). For the G' center ensemble with lower and higher concentrations, E_As of 78 meV and 75 meV, respectively, were found.

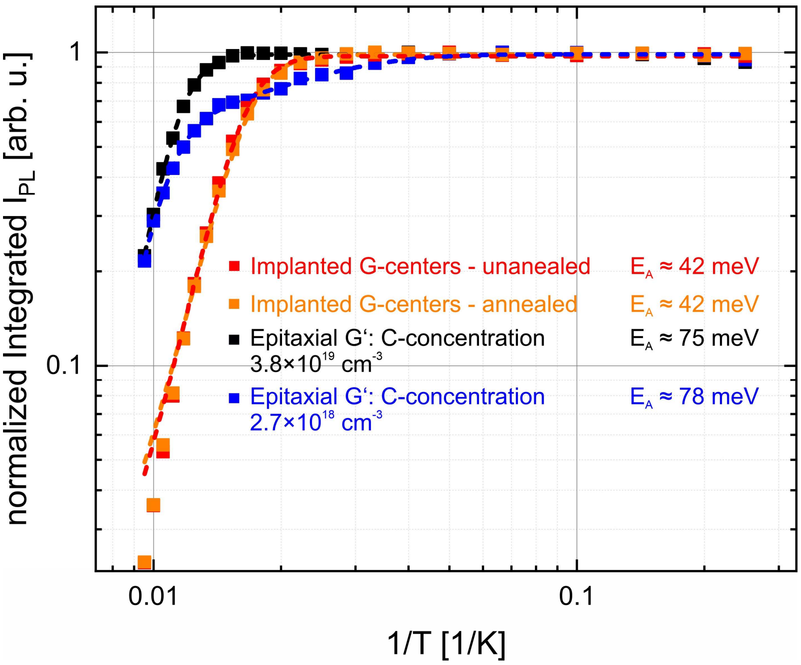

Figure S9: *Arrhenius plots, showing the integrated PL intensity versus inverse PL sample temperature for implanted G-centers and self-assembled G' centers obtained from two different C-concentrations: 3.8×10^{19} cm^{-3} and 2.2×10^{18} cm^{-3}. Dashed lines are fits of the activation energies for the different samples.*

Ab initio **modeling of G'-center: stability and ionization processes**

Our previous results on the calculated optical properties of the G-center and G'-center—where the structure of the former is well established (11)—suggest that the G'-center is a three-carbon atom complex, as shown in Fig. S10a. While calculating the activation energy for decomposing the defect complex that we proposed as our B+C_{Si} model for the G'-center, we recognized that a more detailed study would be required (see our work on G-center in Ref. (12)). However, we observe that the additional C_{Si} completely suppresses the bistability of the pure G-center; that is, the B+C_{Si} configuration is more stable than an A+C_{si} configuration in all charge states (see Figure S10b). Therefore, the G'-center must be more thermally stable, as also indicated by the experiment.

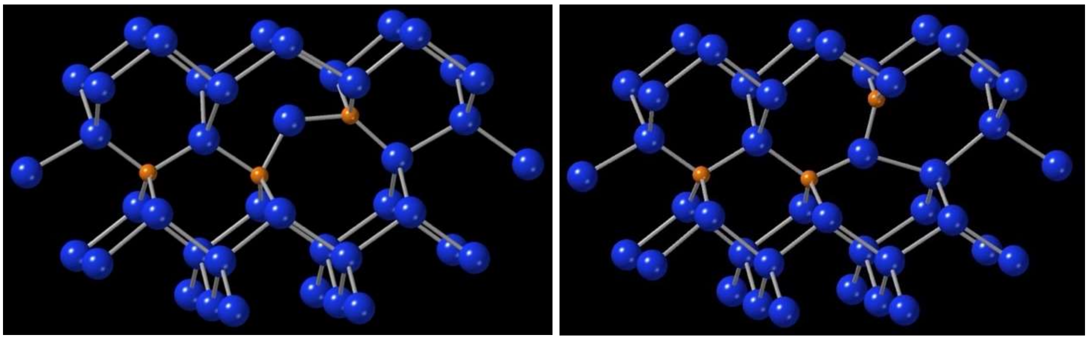

Figure S10: *The B+C_{s1} (a) and A+C_s (b) configurations of the G'-center.*

Next, we investigate the ionization processes of the G-center and G'-center from first principles. It is well known (13) that the G-center is bistable; i.e., it is stable in the B-form as a neutral defect but switches to the A-form once it is ionized, either positively or negatively. The observed ionization acceptor level lies about 80 meV deeper in the band gap (with respect to the conduction band minimum) than the donor level does (with respect to the valence band maximum). As a consequence, the negatively charged G-center plus bound hole system has a lower excited state's energy than the positively charged defect plus bound electron system. Thus, the observed activation energy (E_A) of the G-center can be explained as follows: after neutral excitation, the defect is thermally ionized to the negative charge state by ejecting a hole.

By taking the ionization energy from deep-level transient spectroscopy (DLTS) observations (13), one can estimate the energy gap between the neutral excited state (970 meV) and the ionization threshold energy (1020 meV) as approximately 50 meV. In this estimation, we did not account for the temperature-induced shift of the band edges, which may affect the analysis, as the DLTS measurements were carried out at higher temperatures than the photoluminescence (PL) measurements. Nevertheless, this result strongly indicates that the observed activation energy, $E_A = 42$ meV, for the G-center is associated with this ionization process.

We calculated the acceptor and donor charge transition levels of the G-center (see our previous paper on the G'-center for the calculation methods and parameters used (7); finite-size effects were treated using our developed method (14). The acceptor level indeed lies 40 meV deeper in the band gap than the donor level, which further supports our proposition.

We then calculated the donor and acceptor levels of the G'-center. We found that both levels shift closer to the band edges compared to those of the G-center. Most importantly, the calculated acceptor level shifts 20 meV higher in the band gap. By combining the calculated ZPL energies (see Ref. (7) and the acceptor levels relative to the valence band maximum, we obtain $\Delta E_A = 34$ meV between the G'-center and the G-center, which agrees well with the observed values of 35–38meV.

Our results explain the experimental observation that ΔE_A is larger than the energy difference between the ZPL energies of the G-center and G'-center: the ionization energy of the ground state of the G'-center is larger than that of the G-center. This agreement further reinforces our model of the G'-center.

References

1. Yabumoto, N. Analysis of molecular adsorbates on Si surfaces with thermal desorption spectroscopy. *AIP Conf. Proc.* **1998,** 449, 696–701.
2. Wind, L.; Sistani, M. ; Böckle, R.; Smoliner, J. ; Vukũsić, L. ; Aberl, J. ; Brehm, M.; Schweizer, P.; Maeder, X.; Michler, J.; Fournel, F.; Hartmann, J.-M.; Weber, W. M. Composition Dependent Electrical Transport in $Si_{1-x}Ge_x$ Nanosheets with Monolithic Single-Elementary Al Contacts. *Small* **2022,** 18(44), 2204178.
3. Wilflingseder, C.; Aberl, J.; Navarrete, E. P.; Hesser, G.; Groiss, H.; Liedke, M. O.; Butterling, M.; Wagner A.; Hirschmann, E.; Corley-Wiciak, C.; Zoellner, M. H.; Capellini, G.; Fromherz, T.; Brehm, M. Ge Epitaxy at Ultralow Growth Temperatures Enabled by a Pristine Growth Environment, ACS Appl. Electron. Mater. **2024**, https://doi.org/10.1021/acsaelm.4c01678
4. Fuchsberger, A.; Wind, L.; Sistani, M.; Behrle, R.; Nazzari, D.; Aberl, J.; Navarrete, E. P.; Vukũsić, L.; Brehm, M.; Schweizer, P.; Vogl, L.; Maeder, X.; Weber, W. M. Reconfigurable Field-Effect Transistor Technology via Heterogeneous Integration of SiGe with Crystalline Al Contacts. *Advanced Electronic Materials* **2023,** 2201259.
5. Salomon, A.; Aberl, J.; Vukušić, L.; Navarrete, E. P.; Marböck, J.; Enriquez, D.-H.; Schuster, J.; Martinez, K. G. H.; Groiss, H.; Fromherz, T.; Brehm, M. A group-IV double heterostructure light emitting diode for room temperature gain in Silicon, 2024, https://doi.org/10.48550/arXiv.2409.11081 (accessed November 25, 2024)
6. Salomon, A.; Aberl, J.; Vukušić, L.; Hauser, M.; Fromherz, T.; Brehm, M. Relaxation delay of Ge-rich epitaxial SiGe films on Si (001). *physica status solidi (a)* **2022,** 219, 2200154.
7. Aberl, J.; Navarrete, E. P.; Karaman, M.; Enriquez, D. H.; Wilflingseder, C.; Salomon, A.; Primetzhofer, D.; Schubert, M. A.; Capellini, G.; Fromherz, T.; Deák, P.; Udvarhelyi, P.; Li, S.; Gali, Á.; Brehm, M. All-Epitaxial Self-Assembly of Silicon Color Centers Confined Within Sub-Nanometer Thin Layers Using Ultra-Low Temperature Epitaxy, *Advanced Materials* **2024**, 36 (48), 2408424.
8. Redjem, W.; Durand, A.; Herzig, T.; Benali, A.; Pezzagna, S.; Meijer, J.; Yu Kuznetsov, A.; Nguyen, H. S.; Cueff, S.; Gérard, J.-M.; Robert-Philip, I.; Gil, B.; Caliste, D.; Pochet, P.; Abbarchi, M.; Jacques, V.; Dréau, A.; Cassabois G. Single artificial atoms in silicon emitting at telecom wavelengths, *Nature Electronics* **2020,** 3, 738.
9. Davies, G. The optical properties of luminescence centres in silicon, Phys. Rep. 176, 83 (1989); https://doi.org/10.1016/0370-1573(89)90064-1
10. Beaufils, C.; Redjem, W.; Rousseau, E.; Jacques, V.; Kuznetsov, A. Y.; Raynaud, C.; Voisin, C.; Benali, A.; Herzig, T.; Pezzagna, S.; Meijer, J.; Abbarchi, M.; Cassabois, G.; Optical properties of an ensemble of G-centers in silicon, *Phys. Rev. B* **2018**, 97, 035303.
11. Udvarhelyi, P.; Somogyi, B.; Thiering, G.; Gali, A. Identification of a Telecom Wavelength Single Photon Emitter in Silicon, *Phys. Rev. Lett.* **2021**, 127, 196402.

12. Deák, P.; Udvarhelyi, P.; Thiering, G.; Gali, A. The kinetics of carbon pair formation in silicon prohibits reaching thermal equilibrium. *Nat. Commun.* **2023,** 14, 361.
13. Song L. W.; Zhan, X. D.; Benson, B. W.; Watkins, G. D. Bistable interstitial-carbon–substitutional-carbon pair in silicon, *Physical Review B* **1990**, 42, 5765.
14. da Silva, M. C.; Lorke, M.; Aradi, B.; Tabriz, M. F.; Frauenheim, T.; Rubio, A.; Rocca, D.; Deák, P. Self-Consistent Potential Correction for Charged Periodic Systems. *Phys. Rev. Lett.* **2021**, 126, 076401.
